# Milk Stones in Dairy Cattle: From Pathogenesis to Ultrasonographic Diagnosis and Clinical Management

**DOI:** 10.3390/ani16213446

**Published:** 2026-11-01

**Authors:** Katarzyna Żarczyńska

**Affiliations:** Department and Clinic of Internal Diseases, Faculty of Veterinary Medicine, University of Warmia and Mazury in Olsztyn, 10-719 Olsztyn, Poland; katarzyna.zarczynska@uwm.edu.pl

**Keywords:** lactolith, mammary gland, ultrasonography, teat obstruction, milk flow, dairy cow, udder health

## Abstract

Milk stones (lactoliths) are poorly understood structures occurring within the ductal and cisternal systems of the bovine mammary gland. Although they have been described in the veterinary literature for more than 150 years, data on their formation, composition, prevalence, and clinical significance remain limited. This review synthesizes evidence identified through searches of PubMed, Scopus, Web of Science, and Google Scholar, supplemented by screening reference lists and historical veterinary sources. Historical concepts of their pathogenesis have included precipitation of milk minerals, aggregation or coagulation of proteins, and the formation of an organic core around which organic and mineral components may subsequently accumulate. Studies of *corpora amylacea*, secretory concretions, and mineralization of alveolar contents confirm that protein–mineral structures can form within the mammary gland; however, their relationship to clinically recognized lactoliths has not been established. Likewise, no direct association has been demonstrated between lactolith formation and prolonged milk accumulation, incomplete emptying of the gland, or mastitis. The clinical significance of lactoliths primarily results from their potential to cause partial, intermittent, or complete impairment of milk outflow. Ultrasonography enables their localization and morphological assessment and assists in differentiating them from other causes of obstruction; however, validated ultrasonographic criteria for the definitive identification of these structures are lacking. This review summarizes current knowledge of the terminology, possible mechanisms of formation, clinical significance, diagnosis, and management of lactoliths in cattle and identifies the most important directions for future research.

## Simple Summary

Milk stones are structures that form in the mammary gland of cows. They may remain unnoticed, but in some cases they can interfere with or prevent milk flow. Historical descriptions and clinical observations indicate that milk stones may differ in appearance and contain both organic and mineral components. However, their precise composition, mechanisms of formation, and clinical significance remain insufficiently understood. The aim of this review was to collect and organize the available information on milk stones in cattle, with particular emphasis on their formation, structure, and diagnosis using ultrasonography, as well as the management of clinical cases. Ultrasonography allows for their detection and localization within the mammary gland. A better understanding of these structures may facilitate their diagnosis, appropriate clinical interpretation, and selection of suitable management in cows in which they interfere with milk flow.

## 1. Introduction

Milk stones, also referred to in the literature as lactoliths or milk calculi, are structures occurring within the ductal and cisternal systems of the bovine mammary gland. These structures have been described in cattle for more than 150 years [1]; however, the available information remains scattered. Historical chemical analyses identified calcium carbonate as the predominant component of certain milk stones, whereas other described concretions contained varying proportions of organic and mineral material [1]. Proposed mechanisms of formation have included precipitation of milk minerals, coagulation of milk proteins, and the accumulation of material around an organic core [1,2,3]. Clinically, lactoliths may vary in size, number, and location, and their clinical significance is primarily related to their potential to interfere with milk outflow from the affected quarter. Depending on their location and size, they may cause intermittent impairment of milk flow or lead to complete obstruction [2,4,5]. Such mechanical obstruction may prevent complete milk removal from the affected quarter [4,5]. However, its long-term consequences for mammary gland function remain insufficiently investigated.

Related mammary concretions have also been described in other animal species. Histological studies have identified corpora amylacea in the mammary glands of goats, sheep, and horses, with similarities in their morphology and distribution to those observed in cattle [6,7]. Secretory concretions containing organic and mineral components have also been investigated in cattle, goats, and sheep [8]. In addition, clinical observations in cattle and buffaloes have described mobile intraluminal masses, including structures identified as lactoliths, associated with impaired milk outflow [9]. However, these findings concern different types of mammary concretions, and their relationship to clinically recognized bovine lactoliths, particularly regarding composition, formation mechanisms, and clinical significance, remains uncertain. Stone-like concretions causing obstruction of nipple ducts have also been described in two breastfeeding women, with elemental analysis identifying calcium and sulfur as the predominant elements. However, the limited number of cases and the absence of comprehensive structural and mineralogical characterization preclude direct comparisons with clinically recognized bovine lactoliths [10].

Ultrasonography plays an important role in the evaluation of lesions within the ductal and cisternal systems of the mammary gland. It enables non-invasive imaging of the teat and milk cisterns and localization of structures within their lumen, including lactoliths [11]. Combined with clinical examination, ultrasonography can be used to determine the location and morphological characteristics of a lesion and to differentiate it from other causes of impaired milk outflow.

Despite the long history of reports on lactoliths, current knowledge of their biology and clinical significance remains limited and is based on a relatively small number of publications. The aim of this review is to organize and critically evaluate the available evidence regarding the terminology, possible mechanisms of formation, clinical significance, diagnosis, and management of lactoliths in cattle, with particular emphasis on ultrasonography, as well as to identify the most important directions for future research.

## 2. Materials and Methods

This article was prepared as a narrative review of the literature on lactoliths in dairy cows, focusing on their potential mechanisms of formation, clinical significance, diagnosis, and management. Relevant literature was identified through searches of PubMed, Scopus, the Web of Science Core Collection, and Google Scholar, supplemented by screening the reference lists of retrieved publications and examining historical veterinary sources.

The search strategy included terms directly related to lactoliths (lactolith, milk stone, milk calculi) and cattle (cattle, bovine, dairy cow). The search was extended to studies addressing mineralization within the mammary gland (*corpora amylacea*, secretory concretions, mammary calcification), milk physicochemistry (casein micelles, calcium phosphate, milk mineral equilibrium), mammary gland inflammation (mastitis), milk retention (milk stasis, milk accumulation), and the diagnosis and treatment of impaired milk outflow (teat obstruction, udder ultrasonography, theloscopy, teat surgery). Search terms were combined using the Boolean operator AND, with combinations adapted to the topic being investigated. The German term Milchsteine was also used to identify historical sources.

Searches using terminology directly related to lactoliths yielded 10 records in Scopus and five in the Web of Science Core Collection. In PubMed, searches restricted to titles and abstracts for “milk stones”, “lactolith”, and “teat calculi” yielded 0, 1, and 0 records, respectively, while the combined search in Google Scholar returned approximately 289 results.

Selected searches addressing related topics yielded the following numbers of records in PubMed, Scopus, and the Web of Science Core Collection, respectively: “*corpora amylacea*” AND “mammary gland” (11, 20, and 26), “casein micelles” AND “calcium phosphate” (128, 368, and 431), mastitis AND “milk calcium” (3, 5, and 3), “teat obstruction” AND ultrasonography AND cattle (0, 0, and 0), “teat obstruction” AND cattle (2, 10, and 7), and theloscopy AND cattle (6, 9, and 7). The corresponding searches in Google Scholar yielded approximately 468, 9200, 509, 79, 196, and 159 results, respectively. These figures represent the numbers of records retrieved for individual search queries and do not account for overlapping results between databases or searches.

Eligible publications included original research articles and clinical case reports concerning lactoliths in cattle, as well as studies providing evidence relevant to evaluating their proposed mechanisms of formation, diagnosis, and treatment. Selected review articles, historical publications, and veterinary textbooks were also considered. No restrictions were imposed on the year of publication. Publications in English, German, and Polish were considered. The literature search was last updated in October 2026.

Retrieved records were initially assessed for relevance based on their titles and, where necessary, abstracts. Records clearly unrelated to the scope of the review were not considered further. Full texts of selected potentially relevant publications were subsequently examined. Publications that did not provide information relevant to the scope of the review, as well as materials whose reliability or cited sources could not be verified, were excluded from further consideration. The selected publications were critically evaluated with regard to study design, methodological limitations, and the extent to which their findings supported the conclusions drawn. Particular attention was paid to distinguishing direct evidence concerning clinically identified lactoliths from indirect evidence derived from studies of other mammary structures and the physicochemical properties of milk.

Following the initial assessment of titles, 153 publications were selected for further evaluation. Of these, 38 were excluded following abstract screening or because the full text was unavailable. The full texts of the remaining 115 publications were examined, of which 60 were not retained because they did not provide information sufficiently relevant to the objectives of the review, did not contribute additional evidence, or largely duplicated information available in other sources. Ultimately, 55 references were included in the review. A meta-analysis was not feasible because the available literature specifically addressing bovine lactoliths consists largely of isolated clinical reports and heterogeneous morphological observations. Differences in study designs, diagnostic methods, and reported outcomes, together with the lack of comparable quantitative data, precluded meaningful statistical pooling.

## 3. Terminology, Definition, and Anatomical Location of Lactoliths

In the veterinary literature, milk stones have been described using several terms, primarily lactolith, milk stone, and milk calculus. The terms lactolith and milk stone have been used interchangeably in both older and more recent clinical reports. Winkler [2] used the term “milk stones or lactoliths”, whereas Sahoo and Ganguly [5] used both terms to refer to the same lesion. A universally accepted definition of a lactolith has not yet been established. For the purposes of this review, a lactolith is provisionally defined as a discrete, compact concretion occurring within the ductal or cisternal system of the bovine mammary gland, either freely movable or attached to the mucosa. This operational definition is based on the morphological descriptions available in the literature and does not imply a specific mineral composition or mechanism of formation.

In the classical description, milk stones were characterized as compact, oval, grayish-red structures approximately 5 mm in diameter, containing both organic and inorganic material [2]. They may occur singly or as multiple structures; most commonly, one or two lactoliths per quarter have been reported, although the presence of 25–30 structures has also been documented [2]. Much larger, hard lactoliths requiring surgical removal have also been described [5], indicating a broad spectrum of possible sizes and macroscopic characteristics. Historical analyses indicated that structures described as “true milk stones” (German: wahre Milchsteine) consisted mainly of calcium carbonate, whereas other types of concretions differed in their proportions of mineral and organic components [1]. However, comprehensive characterization of the composition of bovine lactoliths using modern analytical methods remains limited. Therefore, no specific size, consistency, or mineral composition can currently be considered a defining feature of a lactolith.

Available clinical reports primarily concern lactoliths located in the teat cistern, where they may be palpable and, depending on their size and location, interfere with milk outflow [11,12]. However, the location of lactoliths does not appear to be restricted to the teat cistern. Winkler [2] described their formation within the ductal system and subsequent movement toward the teat with the onset of lactation. Large lactoliths located more proximally within the udder have also been described, although their precise anatomical location was not specified [3]. Lactoliths located in the gland cistern have been reported much less frequently, and data concerning this location remain limited.

## 4. Current Concepts of Pathogenesis

Historical descriptions provide evidence of mineral and organic components in milk stones [1,2,3], while experimental and morphological studies have established important aspects of milk protein–mineral interactions [13,14,15,16] and the composition and morphology of other mammary concretions [6,7,8]. However, these findings do not establish the mechanisms responsible for the formation of clinically recognized bovine lactoliths. The following sections distinguish established findings from proposed mechanisms and examine the potential roles of milk stasis and inflammation.

### 4.1. Historical Hypotheses on Lactolith Formation

The first detailed concepts regarding the formation of milk stones date back to the nineteenth century. Early descriptions indicated the presence of several types of concretions within the mammary gland, differing in structure and composition. In structures referred to as “true milk stones”, a mineral core was described, around which further deposition of mineral salts occurred. Chemical analyses showed that calcium carbonate was the predominant component of these structures. Prolonged retention of milk in the cistern and ducts was suggested as one of the factors promoting mineral precipitation. A different mechanism was proposed for “pseudo-milk stones” (German: Pseudomilchsteine), in which a casein clot was considered the starting point for the subsequent deposition of mineral components [1]. Later descriptions continued to propose two main mechanisms: coagulation of milk proteins and precipitation of milk minerals. Concretions formed from coagulated casein were associated, among other factors, with inflammation of the mammary gland, whereas others were interpreted as accumulations of mineral salts derived from milk [3]. A subsequent concept placed greater emphasis on the presence of material that could serve as a core for a developing lactolith. Irritation of the epithelium of the ductal system, particularly during the dry period, was thought to result in cell desquamation, followed by the deposition of organic and inorganic components around these cells. Microorganisms or fluids retained within the ductal system were suggested as potential factors initiating this process. The occurrence of milk stones was also associated with infections already present at dry-off, new infections during the dry period, and the intramammary administration of irritating preparations [2].

Historical hypotheses of lactolith formation therefore involved three main elements: precipitation of milk minerals, aggregation or coagulation of proteins, and the presence of material that could serve as a core for the developing concretion. Milk stasis and inflammation of the mammary gland were suggested as potential factors promoting these processes. However, these historical hypotheses were based primarily on clinical observations and early chemical analyses, and the proposed mechanisms have not been directly demonstrated in studies of clinically recognized bovine lactoliths.

### 4.2. Milk Protein–Mineral Interactions

Milk is a complex protein–mineral system in which calcium and phosphate are present both in the soluble phase and in association with casein micelles. Within casein micelles, calcium phosphate occurs as nanoclusters of amorphous calcium phosphate (ACP), stabilized by caseins. Sequestration of calcium phosphate allows high concentrations of calcium and phosphate to be maintained in milk without their spontaneous precipitation as insoluble calcium phosphates [13,14]. The ability of phosphorylated casein sequences to stabilize calcium phosphate as amorphous nanoclusters and prevent macroscopic precipitation was demonstrated experimentally using a β-casein-derived phosphopeptide in a model solution [15]. The stability of this system depends on the interactions between casein and mineral components and on the physicochemical conditions of milk. Holt [16] developed an equilibrium thermodynamic model demonstrating how the partitioning of calcium phosphate between soluble and micellar phases depends on the availability of phosphorylated casein binding sites. Application of this model to published milk composition data suggested a greater susceptibility to calcium phosphate precipitation in some samples obtained during late lactation and mastitis. However, these findings represent theoretical predictions of mineral instability rather than direct evidence of pathological concretion formation. Furthermore, the model relies on assumptions concerning calcium phosphate nanocluster composition and the behavior of casein phosphate centers, which should be considered when interpreting its biological implications. Experimental evidence that caseins and calcium phosphate can form mixed precipitates under specific physicochemical conditions was provided by Guo et al. [17], who observed co-precipitation in model systems containing purified β-casein or commercial caseinates, calcium ions, and phosphates. Although these experiments demonstrate the possibility of protein–mineral co-precipitation, neither this process nor the mineral instability predicted by thermodynamic models has been directly linked to the formation of clinically recognized lactoliths in cattle.

Genetic variants of caseins, particularly κ-casein, may influence the structural properties of casein micelles. Associations between κ-casein variants, glycosylation, and micelle size have been demonstrated in bovine milk [18,19]. However, these structural differences do not necessarily correspond to changes in micellar mineralization. Although significant differences in micelle size have been observed among κ-casein genotypes, no significant association with the degree of casein mineralization, expressed as protein-associated calcium per unit of casein, was demonstrated [19]. Other studies have likewise found no significant associations between the investigated milk protein variants and the distribution of calcium and phosphorus between milk fractions or between κ-casein genotypes and casein-associated calcium and phosphorus [20,21]. Thus, although casein polymorphisms are associated with differences in micellar structure, their contribution to calcium phosphate precipitation or the formation of clinically recognized lactoliths has not been established.

### 4.3. Mammary Concretions and Mineralization

Beyond studies of milk physicochemistry, morphological investigations of *corpora amylacea* provide evidence of protein–mineral concretion formation within the bovine mammary gland. These structures are not equivalent to clinically recognized lactoliths; however, ultrastructural studies have allowed for a sequence of successive stages in their development to be proposed. According to the proposed ultrastructural model, the process begins within the alveolar lumen with the aggregation, fusion, and gradual compaction of casein micelles. The resulting compact structure forms a core, onto the surface of which additional material of micellar origin is subsequently deposited. Further growth occurs by accretion, resulting in an increasingly complex, concentrically layered structure [22]. As *corpora amylacea* increase in size, the proportion of mineral material may also increase. Larger structures contained substantial amounts of crystalline material, and the presence of mineral components was confirmed histochemically. As a possible explanation for these observations, it was proposed that fusion of micelles may be followed by separation of their organic and inorganic components and crystallization of calcium phosphate, while the organic matrix derived from casein remains part of the developing structure. However, this mechanism should be regarded as an interpretation of the observed mineralization rather than a directly demonstrated sequence of chemical transformations [22]. The complex protein–mineral nature of *corpora amylacea* is further supported by subsequent studies. Amyloid, calcium deposits, and glycoproteins have been identified in these structures, and their location suggests that nucleation may occurs primarily within the alveolar lumen [23]. Compositional analyses have also demonstrated the presence of milk proteins, including caseins, β-lactoglobulin, and α-lactalbumin, together with substantial amounts of calcium and phosphorus [24]. Taken together, these observations confirm that structures containing both organic components of milk origin and a mineral component can form within the bovine mammary gland. The occurrence of *corpora amylacea* was not significantly associated with intramammary infection status or somatic cell count. Most structures were located within the alveolar lumen. Based on morphological observations, Nickerson et al. [25] proposed that their accumulation might obstruct alveolar lumina and small ducts, potentially contributing to milk stasis and local mammary involution. However, this proposed mechanism was not directly demonstrated. Similarly, ultrastructural studies provided no evidence that mastitis is a prerequisite for their formation [22].

Studies of secretory concretions of the mammary gland provide further information on structures that may reach clinically relevant sizes. These structures consisted of an organic matrix formed from condensed secretion, with mineral material deposited within it. Smaller concretions showed a concentrically layered organization, whereas larger ones could form agglomerates of smaller structures. In partially mineralized concretions, mineral material was located predominantly in the center, while successive non-mineralized layers of condensed secretion were present at the surface. Concretions from cows were also shown to contain bone sialoprotein, osteocalcin, osteonectin, and keratan sulfate. In contrast, osteopontin was not detected by immunohistochemistry in the examined secretory concretions [8]. Kumura et al. [26] demonstrated that osteopontin isolated from bovine milk inhibited calcium phosphate precipitation in a model aqueous system. Whether osteopontin influences mineral deposition within the mammary gland or the formation of clinically recognized lactoliths remains unknown. Secretory concretions do not necessarily remain microscopic. Some may reach a size that allows for clinical detection, and in cattle, hard, mineralized concretions capable of moving into the teat cistern and interfering with milk outflow have been described. However, they were not referred to as lactoliths; therefore, these findings do not justify equating secretory concretions with clinically recognized milk stones [8].

More recent histopathological observations indicate that mineralization of alveolar contents may occur to varying degrees, ranging from single small deposits to massive, compact mineral material filling a substantial portion or the entire alveolar lumen and associated with alveolar dilation and epithelial degeneration [27]. Mean somatic cell count increased with the severity of mineralization; however, this relationship was assessed descriptively only, without formal statistical testing. Therefore, this study does not allow the direction or nature of the relationship between mineralization and local inflammation to be determined.

It has not been established whether *corpora amylacea*, secretory concretions, mineralization of alveolar contents, and clinically recognized lactoliths represent different stages of the same process, partially related phenomena, or distinct lesions. However, the available evidence indicates that complex protein–mineral structures can form within the bovine mammary gland [8,22,23,24]. The formation of an organic core or matrix, followed by its mineralization and further growth, can therefore be considered a potential mechanism of lactolith development; however, such a sequence has not yet been demonstrated in clinically recognized milk stones. The main anatomical, morphological, and compositional characteristics of these structures are summarized in Table 1.

### 4.4. Milk Stasis and Incomplete Mammary Emptying

Milk stasis and incomplete emptying of the mammary gland have long been considered potential factors associated with the formation of milk stones. Historical descriptions suggested that prolonged retention of milk within the cisterns and ducts may promote the formation of concretions [1]. However, this hypothesis has not been directly confirmed. Contemporary studies have shown that milk retention induces local changes within the mammary gland. Prolonging the interval between milkings may increase the permeability of the secretory epithelium as a result of changes in tight junction integrity [28]. Substantial incomplete emptying of the udder also leads to increased filling of the gland cistern and changes in epithelial permeability and the ionic composition of milk, including changes in Na and K concentrations [29]. However, neither study investigated protein aggregation, calcium–phosphate precipitation, mineralization, or lactolith formation; therefore, their relevance to the pathogenesis of lactoliths remains unestablished.

There is, however, a basis for considering the relationship in the opposite direction. *Corpora amylacea* may occupy the lumen of alveoli and small ducts, potentially restricting the outflow of secretion [25], whereas larger secretory concretions may move within the ductal and cisternal systems and mechanically interfere with milk outflow [8]. Milk stasis may therefore be not only a historically proposed factor preceding lactolith formation but also a consequence of the presence of a concretion. However, a direct causal relationship between milk retention and the initiation or growth of lactoliths has not yet been demonstrated.

### 4.5. Mastitis and Local Inflammatory Environment

An association between mastitis and lactolith formation has not yet been demonstrated. Although inflammation of the mammary gland was included in historical concepts of their pathogenesis, there are no studies evaluating the occurrence or development of lactoliths in relation to the presence of intramammary infection, the severity of the inflammatory response, or a history of mastitis. Mastitis is associated with changes in milk composition, including its mineral components. Available studies indicate, among other findings, decreased calcium concentrations and changes in phosphorus and other ions in milk from affected quarters [30,31,32]. Inflammation-associated changes in milk pH may also influence the equilibrium between soluble and micelle-associated calcium phosphate. In an experimental study using bovine milk supplemented with calcium gluconate or calcium lactate, Wang et al. [33] demonstrated that increasing pH was associated with a decrease in calculated free Ca^2+^ and H_2_PO_4_^−^ concentrations, whereas HPO_4_^2−^ concentration changed only slightly. However, these findings were obtained under experimentally modified milk conditions and cannot be directly extrapolated to milk from cows with mastitis.

Furthermore, the relationship between intramammary infection and milk pH is not consistent across studies. Kandeel et al. [34] reported a positive association between milk pH and somatic cell count, which was more pronounced before drying-off than during early lactation, although pH measurements showed limited diagnostic accuracy for detecting subclinical mastitis. Citrate is another important component of milk mineral equilibrium because it complexes calcium ions and influences their availability for calcium phosphate formation [16]. Experimental studies using model aqueous calcium phosphate solutions have demonstrated that citrate inhibits crystal growth and reduces the size and crystallinity of calcium phosphate precipitates [35]. Nevertheless, alterations in citrate concentration cannot be assumed to occur uniformly during intramammary infection. In a study involving clinically healthy dairy cows, Zalewska et al. [36] found no significant differences in either milk pH or citric acid concentration among groups differing in bacteriological status. However, whether these physicochemical changes contribute to the formation of clinically recognized lactoliths remains unknown. Based on the available evidence, mastitis should be regarded as a historically proposed but unconfirmed factor in lactolith formation.

## 5. Clinical Presentation and Consequences for Milk Flow

The clinical significance of lactoliths is primarily related to their potential to interfere with milk outflow from the affected quarter. Structures located within the ductal and cisternal system may mechanically impede emptying of the gland, and the nature of the resulting impairment depends, among other factors, on their location and mobility. During milking, a lactolith may move from the gland cistern into the teat cistern and, depending on its position, intermittently restrict or completely block milk flow. Consequently, obstruction may be partial, intermittent, or complete [2,4,9].

The mechanical effect of a lactolith may be particularly pronounced when it moves into the distal part of the teat cistern. Classical descriptions indicate that a mobile stone may act as a valve, blocking milk outflow [2]. Marked impairment of milk outflow may also occur with large lactoliths located more proximally within the udder. In the cow described by Sahoo and Ganguly [5], there was a complete absence of milk flow from the affected part of the gland, which persisted even after insertion of a teat siphon. The large, hard structures were removed through a surgical incision on the lateral aspect of the udder.

In addition to impaired milk outflow, mammary gland concretions may be associated with local discomfort. Egerbacher et al. [8] described mild to moderate teat pain and uneasy behavior in cows with palpable mineralized concretions. The authors also suggested that impaired milk drainage and subsequent milk stasis may predispose the mammary gland to inflammation.

Although the long-term consequences of lactolith-associated impairment of milk outflow have not been investigated, experimental studies indicate that prolonged incomplete milk removal may affect mammary secretory function. Deacon et al. [37] demonstrated that incomplete milking was associated with reduced expression of genes involved in milk synthesis, decreased milk lactose concentration, and reduced mammary gland functional capacity. These findings suggest that persistent impairment of milk outflow could have physiological consequences beyond mechanical obstruction.

The available observations therefore indicate that the clinical consequences of lactoliths may range from intermittent impairment of milk flow to complete obstruction. However, the relationship between lactolith characteristics and the severity of milk outflow impairment remains unclear, and the long-term effects of lactolith-associated obstruction on mammary gland function have not been established.

## 6. Diagnosis

### 6.1. Clinical Examination

Clinical examination in a cow with a suspected lactolith should include visual inspection and careful palpation of both the teat and the udder, as well as assessment of milk flow during manual milking. On palpation, lactoliths may be detected as hard structures varying in mobility and location. Well-defined, mobile structures have been described within the teat cistern [4,12], whereas large lactoliths located more proximally may be palpable as hard masses within the udder [5].

The presence of a lactolith is not necessarily associated with pain. Acquired lesions causing obstruction of the teat cistern are usually non-painful, although pain may occur in cases of concurrent recent trauma [4]. Examination should therefore assess not only the presence, consistency, and mobility of a palpable structure, but also the condition of the teat and surrounding tissues.

Palpation has important limitations, however, and the ability to detect a lactolith depends on its size and location. When a lesion cannot be palpated or cannot be adequately characterized by palpation, ultrasonography may provide additional information.

### 6.2. Ultrasonography

Ultrasonography is a particularly useful adjunct to clinical examination in cows with a suspected lactolith because it allows for the location of the lesion to be determined and enables the assessment of structures that are non-palpable or difficult to evaluate by palpation. The examination allows for visualization of the teat canal, Fürstenberg’s rosette, the teat cistern, and the gland cistern, as well as lesions located within these structures [11,38,39]. The usefulness of ultrasonography in the diagnosis of lesions causing teat obstruction has also been demonstrated in clinical studies, with the technique being particularly useful for lesions located at the base of the teat [40].

High-frequency transducers, most commonly 7.5–10 MHz, are used for examination of the teat. The examination may be performed by direct application of the transducer to the skin or using the water-bath technique, which reduces deformation of the teat and facilitates assessment of its distal portion. The gland cistern and udder parenchyma are examined transcutaneously by placing the transducer directly on the udder skin [11,39,41]. The cisterns are best evaluated before milking, as the presence of milk facilitates visualization of their lumen.

Milk filling the cistern provides an essentially anechoic background, although in-dividual small echogenic particles may be visible within the lumen [39]. Against this background, a lactolith appears as a distinctly echogenic structure within the cisternal lumen. Franz et al. [11] presented a free lactolith located within the teat cistern and indicated that ultrasonography allows for determination of the presence, location, and size of such a structure. Distal acoustic shadowing has not been described as a consistent ultrasonographic feature of lactoliths, and its absence should not exclude the diagnosis. Acoustic shadowing results from substantial attenuation of the ultrasound beam, including reflection and absorption. Importantly, echogenicity and attenuation are not necessarily correlated; therefore, an echogenic structure may not produce a pronounced distal acoustic shadow [42]. However, the acoustic properties of bovine lactoliths have not been systematically characterized, and the absence of shadowing cannot be attributed to a specific composition.

**Figure 1:**
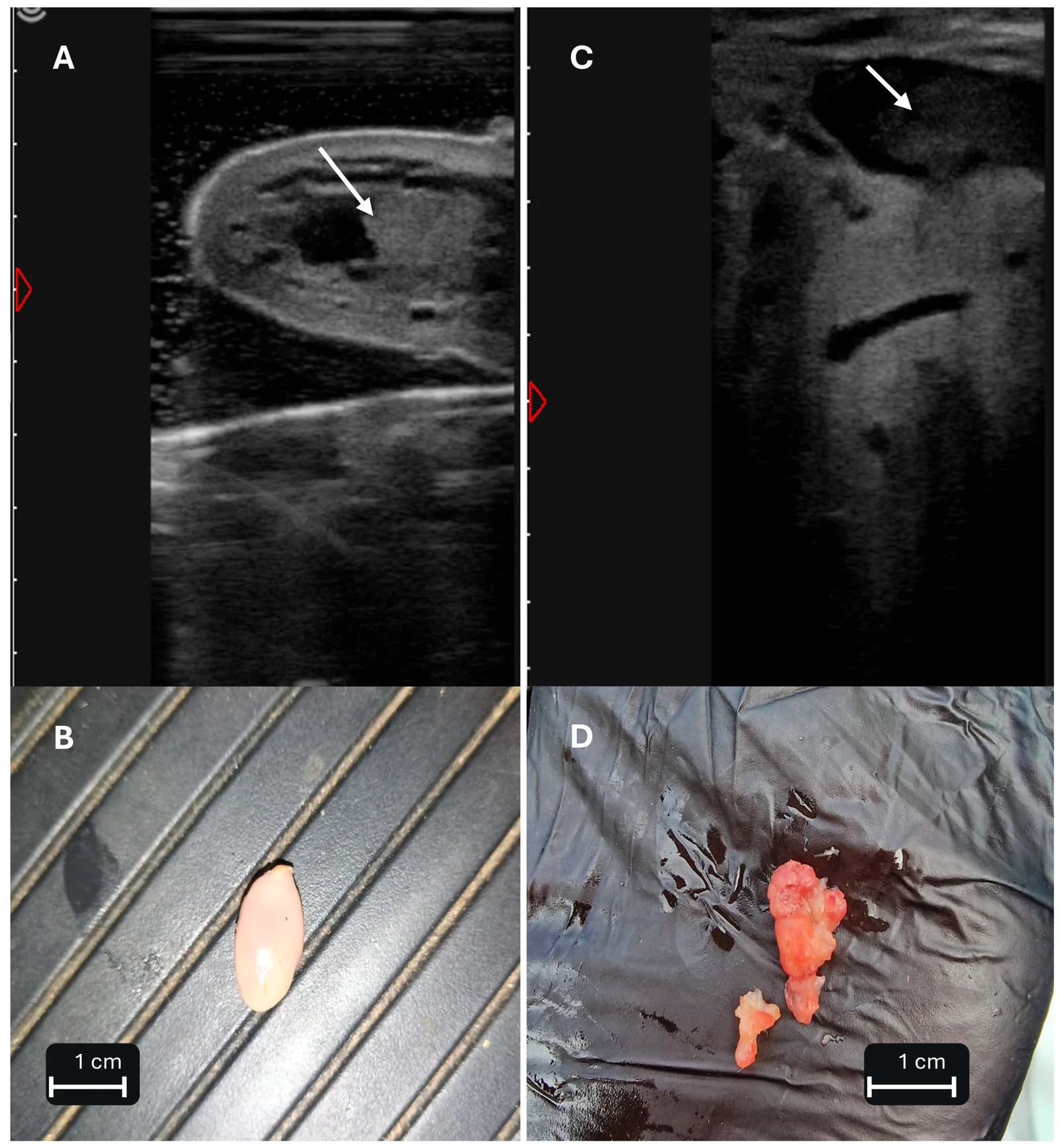
Different ultrasonographic and macroscopic appearances of clinically recognized lactoliths in two cows. (**A**) Lactolith visible as an echogenic structure within the lumen of the teat cistern in cow 1 (arrow). (**B**) Lactolith removed from the teat cistern of the same cow—a compact, smooth, well-defined structure. (**C**) Irregular echogenic structure within the lumen of the gland cistern in cow 2 (arrow), without distal acoustic shadowing. (**D**) Irregular fragments of a clinically recognized lactolith removed through the teat canal from the same cow. Scale bars in panels (**B**,**D**) indicate 1 cm. The extracted structures were not retained for further analysis; therefore, their elemental composition and mineral phases were not determined. Ultrasonographic images were obtained in B-mode at an imaging depth of 12 cm using a 4Vet Slim ultrasound system (Dramiński S.A., Olsztyn, Poland) with an 8 MHz linear rectal transducer; the teat was examined using the water-bath technique, whereas the gland cistern was examined by direct application of the transducer to the skin. Images from the author’s clinical material.

Data on the ultrasonographic appearance of lactoliths in cattle are very limited and relate primarily to lesions located within the teat. Lactoliths located within the gland cistern have been characterized to a much lesser extent, although this location is also accessible to ultrasonographic examination. Examples of the different ultrasonographic and macroscopic appearances of clinically recognized lactoliths are presented in Figure 1. The extracted structures were not retained for further analysis, and their elemental composition and mineral phases were therefore not determined. These clinical observations illustrate a broader limitation of the available literature, in which the identification of lactoliths is rarely supported by detailed compositional analysis.

The presence of an echogenic structure within the cisternal lumen alone is not sufficient to diagnose a lactolith. Ultrasonographic assessment should take into account its location, size, shape, and mobility, as well as its relationship with the cisternal wall. Ultrasonography also enables the simultaneous assessment of mucosal lesions, tissue proliferations, and other intracisternal structures that may interfere with milk outflow and require differentiation from a lactolith [11].

### 6.3. Differential Diagnosis of Teat Obstruction

Acquired teat obstructions may result from proliferation of granulation tissue, mucosal injury, fibrosis, and scarring, particularly following previous trauma. These lesions may be focal or diffuse and may cause varying degrees of impairment of milk outflow [4].

On ultrasonographic examination, proliferative lesions of the mucosa are among the main findings that may require differentiation from a lactolith. Polyps and papillomas, as well as proliferative lesions associated with narrowing of the proximal part of the teat, have been described. These structures are usually connected to the cisternal wall, although in pedunculated lesions the site of attachment to the mucosa may be difficult to visualize [11]. In human breast ultrasonography, Doppler assessment of intraductal vascularity has been investigated as a means of distinguishing papillomas from retained secretions. However, conventional color Doppler imaging has shown limited sensitivity for detecting vascularity in small intraductal lesions, and the applicability of this approach to teat lesions in cattle remains unestablished [43]. Lesions involving Fürstenberg’s rosette and the teat canal should also be considered in the differential diagnosis of obstruction, as should congenital membranes extending across the lumen of the teat cistern, particularly in primiparous cows [4,11].

A separate group comprises free structures within the cisternal lumen, more broadly referred to as floaters. This term includes not only lactoliths but also detached fibrous or granulation tissue within the cisternal lumen. These structures may move within the ductal and cisternal system and interfere with milk outflow; therefore, floater is not synonymous with lactolith [4]. Other intracisternal structures, including coagulated intracisternal material associated with mastitis and blood clots, should also be considered in the differential diagnosis. Ultrasonographic studies have documented coagulated material causing teat obstruction in cows with mastitis [44], while a blood clot presenting as an echogenic intracisternal structure has been reported in a goat [45].

Ultrasonographic assessment should include real-time evaluation of the mobility of the structure and its attachment to the cisternal wall. Ultrasonography allows the location and extent of lesions causing obstruction to be determined [11,40,46]. If the nature of the obstruction remains unclear, additional information may be obtained by teat endoscopy (theloscopy), which allows for direct examination of the teat lumen and its mucosa [47].

The differential diagnosis of intraluminal teat lesions is primarily based on their morphological characteristics, as different lesions may share clinical and ultrasonographic features. The principal distinguishing features and reported management approaches are summarized in Table 2.

## 7. Clinical Management

The aim of managing lactoliths is to restore normal milk outflow while minimizing damage to the teat canal, Fürstenberg’s rosette, and the cisternal mucosa. The choice of intervention should primarily take into account the size and location of the lesion and whether it can be removed safely. Reported management approaches for lactoliths and other intraluminal teat lesions are summarized in Table 2. There are no studies comparing treatment methods for lactoliths, and the available information is derived mainly from individual case reports and from the literature concerning the management of free structures and other mechanical causes of impaired milk outflow.

Small, freely movable structures within the cistern may be removed through the teat canal. Free bodies may be removed after dilation of the teat canal, and some may be expelled with the milk during milking [4]. Such procedures should be performed as atraumatically as possible, because manipulation of the teat canal and mucosa may result in tissue injury, scarring, and secondary stenosis.

For larger structures or lesions that cannot be safely removed through the teat canal, theloscopy may be useful, allowing for direct visualization of the obstruction and intervention under visual control. This technique has been used in the diagnosis and treatment of various mechanical causes of impaired milk outflow [47,50]. Its applicability, however, depends on the location and nature of the lesion and the availability of appropriate equipment.

Large structures located more proximally may require a more invasive approach. Sahoo and Ganguly [5] described a case of large, hard lactoliths associated with complete obstruction of milk outflow that were surgically removed through an incision on the lateral aspect of the udder. This single case report demonstrates that such an approach is feasible but does not assess its safety, indications, or long-term outcomes.

## 8. Knowledge Gaps and Future Research

The most important directions for future research include the definition and classification of lactoliths and the characterization of their composition and origin. Clear criteria for determining which structures should be classified as lactoliths are lacking. It is necessary to establish whether clinically recognized milk stones represent a homogeneous group of lesions or comprise structures of different origin and composition. Contemporary characterization of the mineral, protein, and histological composition of clinically recognized lactoliths is needed. Such analyses could also help determine whether *corpora amylacea*, secretory concretions, mineralization of alveolar contents, and clinical lactoliths represent related phenomena or distinct processes [8,22,27]. The potential contribution of genetic variation in milk proteins, particularly caseins, to susceptibility to lactolith formation also remains unknown. Further research is needed to determine whether specific milk protein variants are associated with lactolith occurrence and whether such associations could be explained by differences in casein micelle structure or mineral interactions.

Another important area concerns the mechanisms of lactolith initiation and growth, as well as their natural history. The local conditions leading to lactolith formation have not been established, nor is it known whether their development begins with an organic core or matrix that subsequently undergoes mineralization. The potential roles of prolonged milk accumulation, incomplete emptying of the gland, and mastitis require particular clarification, without assuming a causal relationship. Data on the rate of growth, changes in morphology, and movement of lactoliths within the ductal and cisternal system are also lacking. Longitudinal studies using serial ultrasonography could allow these changes to be followed in the same animal over time.

Further research is needed to establish validated ultrasonographic criteria for differentiating lactoliths from other intracisternal structures and to determine their prevalence and potential risk factors. Prospective comparison of ultrasonographic features with macroscopic, histological, and chemical characterization of removed material could determine the diagnostic value of features such as size, shape, echogenicity, mobility, acoustic shadowing, and location, particularly for lesions located within the gland cistern. Epidemiological studies determining the prevalence of lactoliths in dairy cow populations are lacking. Prospective studies should consider, among other factors, age, parity, stage of lactation, history of mastitis, and factors related to milking and mammary gland emptying. Identification of true risk factors is essential for the development of evidence-based prevention strategies.

Finally, the clinical significance of lactoliths and the outcomes of different treatment approaches require further investigation. The relationships between lactolith size, number, location, and mobility and impaired milk outflow, milk production, and long-term function of the affected quarter need to be determined. It also remains unknown whether impaired milk outflow and the resulting milk retention increase the risk of intramammary infection. Data comparing the effectiveness of different methods of lactolith removal, recurrence and complication rates, and subsequent function of the affected quarter are also needed.

## 9. Conclusions

Lactoliths are poorly understood structures occurring within the ductal and cisternal systems of the bovine mammary gland that may interfere with milk outflow from the affected quarter. Despite their long history in the veterinary literature, the mechanisms underlying lactolith formation and their relationship with other protein–mineral concretions of the mammary gland remain unclear. Ultrasonography enables localization and morphological characterization of these lesions; however, validated criteria for their definitive identification are currently lacking. Combining clinical and ultrasonographic findings with analysis of removed structures will be essential for better defining lactoliths, elucidating their origin, and determining their true clinical significance in dairy cattle.

## Figures and Tables

**Table 1:** Comparison of clinical lactoliths, corpora amylacea, secretory concretions, and alveolar milk calcification in cattle.

Structure	Predominant Location	Morphological Characteristics	Composition and Supporting Evidence
Clinical lactoliths (milk stones)	Ductal and cisternal systems, particularly the teat cistern	Compact concretions of variable size, occurring singly or in multiples; may be mobile and cause partial or complete obstruction of milk outflow	Historical chemical analyses identified calcium carbonate as the predominant component of certain milk stones, while other concretions contained varying proportions of mineral and organic material. These findings have not been systematically reassessed using contemporary analytical methods [1,2,3,5].
*Corpora amylacea*	Predominantly alveolar lumina; occasionally small ducts, epithelium, and stroma	Microscopic, rounded structures with concentric lamellar, dense, or fibrillar organization	Calcium and phosphorus demonstrated by elemental analysis; caseins and whey proteins identified by biochemical methods. Histochemical staining for amyloid-associated material has yielded variable results [22,23,24,25].
Secretory concretions	Predominantly alveolar lumina; occasionally connective tissue	Microscopic concretions with concentric layering and variable degrees of mineralization	Organic matrix containing glycoproteins and mineral deposits, with histochemical evidence of calcium, phosphate, and carbonate. Osteocalcin, osteonectin, bone sialoprotein, and keratan sulphate detected immunohistochemically in bovine specimens [8].
Alveolar milk calcification	Lumina of mammary alveoli (acini)	Histologically visible deposits ranging from small isolated foci to compact masses partially or extensively filling the alveolar lumen	Calcified deposits described by routine histopathological examination; detailed mineral-phase and protein composition not established [27].

**Table 2:** Differential diagnosis and reported management of intraluminal teat lesions in cattle.

Type of Lesion	Palpation Findings	Ultrasonographic Appearance	Mobility and Relationship to the Teat Wall	Reported Management
Milk stones (lactoliths)	Hard, palpable intraluminal structures; intermittent obstruction during milking may occur [12].	Echogenic structures within the teat cistern [11].	May occur as free intraluminal structures or remain attached to the mucosa [11].	Small stones may be expelled during milking; larger stones have been removed instrumentally through the teat canal, sometimes under ultrasonographic guidance [11,12].
Polyps and papillomatous lesions	Polyps may be palpable as well-defined, pea-sized structures located at the base or in the middle portion of the teat cistern [48].	Echogenic tissue projections arising from the teat wall; the stalk may be difficult to visualize [11].	Attached to the teat wall, sometimes by a stalk [11,49].	Open surgical excision of polyps [48]; theloscopic removal of papillomatous lesions using forceps [50].
Proliferative and fibrotic stenoses	Palpable thickening of the teat wall and focal or diffuse tissue proliferations may be present [49,51].	Narrowing of the teat cistern by tissue of variable, often increased echogenicity; wall thickening, irregular contours, and echogenic mucosal changes may be observed in the proximal or distal teat [11,49,51,52,53].	Wall-associated lesions that restrict the teat cistern lumen [11,49].	Surgical removal of obstructing tissue, including theloscopically guided electrosurgical resection [49,54]. In selected cases, inflammation-associated narrowing may partially resolve following conservative treatment [51].
Congenital and acquired membranous septa	Fibrous, thread-like structures or localized intraluminal obstruction may be palpable [49,55].	Hyperechoic membranous structures, sometimes appearing as linear septa within the teat cistern and causing narrowing or obstruction; a membrane may separate the gland cistern from the teat cistern [11,49,53,55].	Fixed structures attached to the cisternal wall and extending across the lumen [11,49].	Surgical incision or excision of the membrane, including theloscopically guided electrosurgical resection [49,54].
Blood clots	Insufficient evidence is available to define specific palpation findings in the reviewed publications.	A hematoma within the teat sinus has been described as a potential cause of intraluminal obstruction [52].	A mobile hematoma within the teat sinus has been reported [52]; this observation cannot be generalized to all blood clots.	Insufficient evidence is available regarding the treatment of intraluminal blood clots in the reviewed publications.

## Data Availability

The clinical images supporting this review are included in Figure 1. No additional datasets were generated or analyzed for this article.
